# “Engaged, Burned Out, or Both?” A Structural Equation Model Testing Risk and Protective Factors for Social Workers in Refugee and Homeless Aid

**DOI:** 10.3390/ijerph17020583

**Published:** 2020-01-16

**Authors:** Janika Mette, Swantje Robelski, Tanja Wirth, Albert Nienhaus, Volker Harth, Stefanie Mache

**Affiliations:** 1 Institute for Occupational and Maritime Medicine (ZfAM), University Medical Centre Hamburg-Eppendorf (UKE), 20459 Hamburg, Germany; janika.mette@gmx.de (J.M.); swantje.robelski@gmx.de (S.R.); harth@uke.de (V.H.); 2 Competence Centre for Epidemiology and Health Services Research for Healthcare Professionals (CVcare), University Medical Centre Hamburg-Eppendorf (UKE), 20246 Hamburg, Germany; t.wirth@uke.de (T.W.); a.nienhaus@uke.de (A.N.); 3 Department of Occupational Medicine, Hazardous Substances and Public Health, Institution for Statutory Accident Insurance and Prevention in the Health and Welfare Services (BGW), 22089 Hamburg, Germany

**Keywords:** personal burnout, resilience, social work, working conditions, work engagement

## Abstract

The present study sheds light on social workers’ working conditions in highly demanding settings and examines the associations between their perceived job demands, resources, resilience, personal burnout, and work engagement. A cross-sectional quantitative online survey was conducted with employees in social work institutions of independent and public sponsors providing help for refugees and homeless persons. The study participants were 243 social workers (68.8% female and 31.3% male) from four federal states in Germany. Correlations between social workers’ job demands, resources, burnout, and work engagement were confirmed in accordance with the Job Demands–Resources model. Results of the structural equation modelling revealed significant positive effects of employees’ job demands on their personal burnout, but no significant effects on their work engagement. The meaning of work as a job resource was significantly positively related to work engagement and negatively related to burnout. Although resilience did not moderate the relationship between employees’ job demands and burnout, it had a significant negative effect on burnout and a positive effect on work engagement. The results indicate a need for the development of health promotion measures for social workers in homeless and refugee aid. Structural approaches should target the reduction of employees’ job demands to diminish their potentially health-depleting effects. Of equal importance, behavioural measures should foster employees’ meaning of work and resilience, since both resources showed beneficial effects on their work engagement and were negatively related to burnout.

## 1. Background

Social workers offer support and counselling for various groups of clients with regard to the prevention, reduction, and coping with social problems. Their tasks mainly involve person-related services, such as consultancy, education, training, and representation [1]. Two increasingly important areas of social work are refugee and homeless aid. The growing number of people being forcibly displaced represents a social problem with a worldwide scope. According to recent estimates, there are currently 25.9 million refugees worldwide [2]. Additionally, it is estimated that about 100 million people are without a place to live [3]. Although there are no official records available for Germany, data provided by the German Federal working group for homeless aid show that there were about 650,000 homeless people in Germany in 2017, of which about 375,000 people had been recognized refugees [4]. Due to domestic migration and the influx of refugees, there are currently about 1.5 million refugees and asylum seekers in Germany [5].

Both homeless people and refugees are groups of persons with increased vulnerability. As recent studies show, social workers serving homeless individuals and refugees experience several job demands, such as the confrontation with a bureaucratic system, high caseloads, experience of prejudices from other persons against their clients, as well as clients’ suffering from traumatic experiences. The latter can also become apparent during counselling services and thereby affect social workers’ daily working tasks or even cause secondary traumatization [6]. However, social workers described several job resources, too. These primarily referred to the meaning of their work as well as social support from colleagues [7,8].

The present study expands upon existing results by addressing the question how social workers’ job demands (quantitative and emotional demands) and resources (meaning of work and social support) as well as personal resources (resilience) are related to work-related outcomes (work engagement) and strain reactions (personal burnout). The variables were chosen based on previous research findings demonstrating their particular relevance for social workers in refugee and homeless aid [6,7]. Specifically, a recent qualitative study was able to show that social workers perceived high quantitative and emotional demands and cited social support as a crucial job resource [8].

Quantitative demands can be defined as the extensive and intensive demands inherent to one’s work (e.g., work hours, pace, and load). Positive associations between quantitative demands and workers’ stress levels were found for several occupations [9,10,11,12]. With regard to emotional demands, Steinberg and Figart point out that emotional labour encompasses reading emotions of others as well as managing one’s own and others’ emotions [13]. A meta-analysis by Hülsheger and Schewe revealed substantial associations of emotional labour with indicators of impaired well-being [14]. For example, in one study, a positive association between surface acting (as a form of emotional labour) and emotional exhaustion among health care providers was revealed [15]. The job resource meaning of work is composed of the job meaning, the role meaning and the self-meaning at work [16]. Based on the framework of Rosso et al., the key dimensions for meaning of work include the approach and pursuit attached to work as well as direction of action towards the self or others [17]. Social support as another important job resource describes the availability and quality of helping relationships [18]. Evidence suggests positive associations between social support and workers’ health [19,20,21,22,23] as well as direct positive effects of social support on employees’ strain [22]. Two categories of support systems can be assumed for social workers: formal support via management and supervision as well as informal support provided by colleagues or friends [24]. Work engagement represents a work-related outcome characterized by a positive, fulfilling, work-related state of mind [25]. It is a distinct motivational construct that is related to contextual and individual factors, such as task variety and significance [26]. It consists of three key components (vigour, identification, and absorption) and can be understood as the opposite of burnout [25]. In contrast, burnout has been defined as “a state of physical, emotional and mental exhaustion that results from long-term involvement in work situations that are emotionally demanding” [27] p. 501. Kristensen and colleagues further describe fatigue and emotional exhaustion as the core of burnout [28,29].

Social work is often performed in settings characterised by regular changes, e.g., from recently instituted legislation, policies, and practices [24], and has been found to be highly stressful [30]. In view of this, resilience as the ability to adapt to stressors and bounce back from negative experiences plays an important role in social work. In line with the original concept presented by Wagnild and Young, resilience is defined as a trait and personal resource enabling flexible adaption to adverse situations and moderating the effects on negative emotions and stress [31]. Resilience can be understood in terms of two dimensions: (1) personal competences such as self-reliance and independence as well as (2) acceptance of self and life with attributes such as adaptability and flexibility [31,32].

### 1.1. Theoretical Background

The Job Demands–Resources (JD-R) model by Bakker and Demerouti [33,34] may be used in order to explain the underlying mechanisms with regard to the links between social workers’ working conditions, personal, and work-related outcomes. According to the JD-R model, aspects of the job that require physical or mental effort can be considered as job demands, which are positively related to the depletion of health (health impairment process). In contrast, job resources are assumed to reduce job demands and their adverse effects, and to play an important role in increasing employees’ motivation and work engagement (motivational process). The proposed effects of the JD-R model have been empirically proven. Job demands were found to be related to strain reactions and negatively affected employees’ mental and physical health, whereas job resources were associated with positive health effects and employees’ work engagement [35]. 

### 1.2. Study Aims

The aim of our study was to examine the links between social workers’ job demands (quantitative and emotional demands), job resources (meaning of work and social support), personal burnout, and work engagement. Moreover, we aimed to assess the role of resilience as a personal resource in the stressor–strain relationship. 

### 1.3. Hypotheses

Based on the empirical evidence and the JD-R model, the following hypotheses were tested:

**Hypothesis** **1 (H1).** *Quantitative and emotional demands are positively related to social workers’ personal burnout (1a) and negatively related to social workers’ work engagement (1b).*

**Hypothesis** **2 (H2).** *Meaning of work and social support are negatively related to social workers’ personal burnout (2a) and positively related to social workers’ work engagement (2b).*

**Hypothesis** **3 (H3).** *Resilience is negatively related to social workers’ personal burnout (3a) and positively related to social workers’ work engagement (3b).*

**Hypothesis** **4 (H4).** *Resilience moderates the relationship between social workers’ quantitative and emotional demands and personal burnout.*

**Figure 1:**
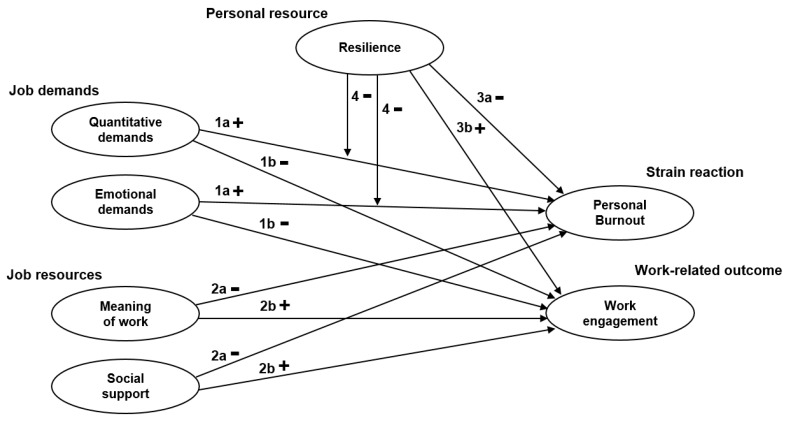
Conceptual model with the hypotheses.

Figure 1 summarizes the relationships between the variables in a conceptual model.

## 2. Materials and Methods

### 2.1. Study Design and Recruitment of Participants

The study was designed as a cross-sectional online survey for social workers in four federal states located in the north/northeast of Germany (Hamburg, Berlin, Schleswig-Holstein, and Mecklenburg–Western Pomerania). Data collection took place between February and May 2019. An extensive internet search was carried out to identify suitable institutions in the refugee and homeless aid. A total of 305 institutions were initially contacted by email and received leaflets with study information. After some days, the institutions were phoned and asked whether they would like to participate in the survey. In sum, 177 institutions refused to participate or were not reached. In contrast, 128 institutions were interested in participation and forwarded the study information to their employees. As inclusion criteria for study participation, social workers had to work in an institution in refugee or homeless aid in one of the four federal states, have direct contact with refugees and/or homeless individuals and be of legal age. All respondents took part in the survey voluntarily. When entering the survey website, they were informed about the study and data confidentiality and gave written consent. The website was visited 298 times. Consent was refused 9 times, 21 people did not start the survey, and 15 people did not complete it. In total, 253 online surveys were included in the data analysis. 

### 2.2. Variables 

#### 2.2.1. Demographic and Workplace Variables

Self-constructed items were used to assess the following variables: gender, age, nationality, federal state, number of inhabitants in place of residence, work area, professional qualification, type of institution, client group, sponsor of institution, professional work experience, working time, type of employment, and management responsibility.

#### 2.2.2. Job Demands and Resources

Scales from the Copenhagen Psychosocial Questionnaire (COPSOQ I) were used to assess social workers’ quantitative demands, emotional demands, social support, and meaning of work [28,36]. The psychometric properties of the COPSOQ I are considered to be good [28,36]. An example item of the scale quantitative demands is “Do you have to work very fast?”, and of the scale emotional demands is “Is your work emotionally demanding?”. An example item belonging to the scale social support is “How often do you get help and support from your colleagues?”. As to the scale meaning of work, an example item is “Is your work meaningful?”. The items were scored on a 5-point Likert scale (1 = always, 5 = never) and transformed to point values ranging from 0 (minimum) to 100 (maximum) for further analyses (e.g., the values 1, 2, 3, 4, and 5 for a 5-response category item were transformed to 0, 25, 50, 75, and 100).

#### 2.2.3. Work Engagement

Work engagement was measured with a 3-item-scale from COPSOQ I [28]. An example item of the scale is “In my work I am full of energy”. The verbal response categories are based on a 5-point Likert scale and transformed to point values from 0 (minimum) to 100 (maximum).

#### 2.2.4. Personal Burnout

Personal burnout was measured with the respective scale from COPSOQ I which is based on the Copenhagen Burnout Inventory [28]. The scale consists of 6 items on a 5-point Likert scale. It was developed to offer the most generic approach to feelings of exhaustion and fatigue regardless of occupational status. An example item of the scale is “How often do you think, ‘I can’t go on’?”. Verbal response categories translate into scoring from 0 (never/to a very low degree) to 100 (always/to a very high degree).

#### 2.2.5. Resilience

A short form of the Resilience Scale—the RS-13—was used to measure social workers’ resilience. The scale focuses on attributes of the core concept, such as emotional stability, optimism, and vitality [31,32]. The RS-13 consists of 13 items based on a 7-point Likert scale (1 = I don’t agree, 7 = I totally agree). An example item of the scale is “When I am in a difficult situation, I usually find a way out”. The psychometric properties of the RS-13 are good [31]. 

### 2.3. Data Analysis 

Statistical analyses were performed with IBM^®^ SPSS^®^ Statistics (version 25) and Analysis of Moment Structures (IBM^®^ SPSS^®^ AMOS™, version 25). There were only 0.5% missing data regarding the variables included in the analysis, justifying the expectation maximization algorithm as a single imputation method [37] to achieve a complete dataset [38]. Data were checked for plausibility and verified for outliers and normality. Although the Shapiro–Wilk test indicated that the data were not normally distributed, skewness and kurtosis of the variables were within the suggested threshold of <1.0 and histograms showed no major deviations from normal distribution. Therefore, parametric tests were used.

Descriptive statistical analyses were conducted to describe associations between the variables. The hypothesized model was tested by means of structural equation modelling (SEM). A confirmatory factor analysis was carried out to assess the reliability, validity, and fit of the measurement model. Linearity was assessed with deviation from linearity tests. Multicollinearity was rejected for all variables [39]. The maximum-likelihood method and bootstrapping with 2000 iterations were used to test the structural model. Work experience was used as a control variable by having it regress on the two endogenous latent variables (burnout and work engagement). Factor scores were imputed from the latent variables to test for moderation. The variables were standardized, and interaction terms were integrated in a path model. To evaluate the goodness-of-fit of the hypothesized model, the following indices were used: χ², χ²/df (ratio of χ² to degrees of freedom), comparative fit index (CFI), root mean square error of approximation (RMSEA) with a 90% confidence interval, and standardized root mean square residual (SRMR). Relatively good model fit was considered based on the following thresholds: CFI ≥ 0.95, RMSEA ≤ 0.06, and SRMR ≤ 0.08 [40]. The statistical significance level was *p* < 0.05. Standardized regressions weights (β) determined the strengths of association between the variables, whereby β = 0.1 was interpreted as a weak, β = 0.3 as a moderate, and β = 0.5 as a strong association [41].

### 2.4. Ethical Considerations

Prior to the interview, all participants signed a declaration of informed consent regarding the performance and recording of the interview. The Medical Ethics Committee of the Hamburg Medical Association provided professional legal and ethical advice for the study (PV5652).

## 3. Results

### 3.1. Characteristics of the Study Population

In total, 68.8% were female and 31.3% were male (Table 1). Most participants were in the age groups of 25–34 years (29.6%) and 35–44 years (27.2%). In sum, 21.8% of the respondents were at least 55 years old. The majority of the participants were qualified as social workers (62.2%). Almost half of the respondents were employed in homeless aid (49.6%), 38.8% worked in refugee aid, and 11.6% in both areas. Around two thirds of the participants (75.1%) worked in institutions with an independent sponsor. Most respondents lived in Berlin (38.3%), followed by Hamburg (37.0%).

### 3.2. Descriptive Analysis

Table 2 displays the characteristics of the variables. Reliability was confirmed for all of them (α > 0.7).

Table 3 depicts the Pearson correlation coefficients. Both job demands were significantly positively related to personal burnout and negatively related to work engagement. The job resources were significantly negatively related to personal burnout and significantly positively related to work engagement. Resilience was significantly negatively related to personal burnout and emotional demands, and significantly positively related to the job resources and work engagement. The correlation between work engagement and personal burnout was significant and negative.

### 3.3. Structural Equation Modelling (SEM)

#### 3.3.1. Confirmatory Factor Analysis (CFA)

The fit of the measurement model was acceptable (χ² = 920,854, df = 570, χ²/df = 1616, *p* < 0.001, CFI = 0.92, RMSEA = 0.05 (0.04–0.06), and SRMR = 0.07). Reliability and validity were confirmed for all included variables (Table 4). The composite reliability (CR) was ≥0.70, the average variance extracted (AVE) was ≥0.50, and the square roots of the AVE (√AVE) were greater than the correlations between the variables. Although the variable Resilience did not entirely reach the recommended thresholds, the deviation was not considered to be large enough to justify exclusion of single items of the well-validated scale.

#### 3.3.2. Structural Model

**Figure 2:**
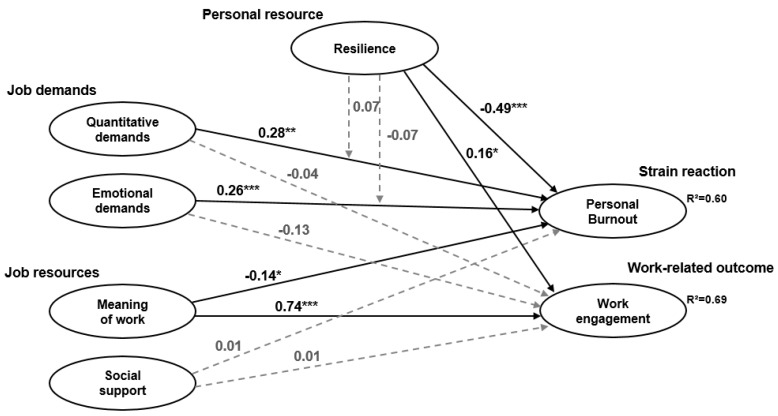
Structural model with standardized path coefficients. Note: χ² = 1001.566, df = 605, χ²/df = 1.655, *p* < 0.001, CFI = 0.91, RMSEA = 0.05 (0.05–0.06), and SRMR = 0.07. Standardized path coefficients are presented on the unidirectional paths. R² = squared multiple correlations. Manifest items, residuals, control variables, and correlations between the variables are not displayed. Non-significant paths are shown in grey and in dotted arrows. * *p* < 0.05; ** *p* < 0.01; *** *p* < 0.001.

Model fit of the structural model was acceptable (χ² = 1001.566, df = 605, χ²/df = 1.655, *p* < 0.001, CFI = 0.91, RMSEA = 0.05 (0.05–0.06), and SRMR = 0.07). The structural model with the standardized estimates of the path coefficients and explained variance for the endogenous variables (personal burnout and work engagement) is presented in Figure 2.

The direct effects of quantitative demands (β = 0.28, *p* < 0.01) and emotional demands (β = 0.26, *p* < 0.001) on social workers’ personal burnout were significant and positive (Table 5), thereby supporting Hypothesis 1a. Moreover, both job demands had negative–but non-significant–effects on work engagement. Therefore, Hypothesis 1b was not entirely confirmed.

Hypotheses 2a and b were partly confirmed as well: as to Hypothesis 2a, meaning of work had a significant and negative effect on personal burnout (β = −0.14, *p* < 0.05), but social support did not. In terms of Hypothesis 2b, meaning of work and social support both had positive effects on work engagement, but only the effect from meaning of work was significant (β = 0.74, *p* < 0.001). 

There was strong support for Hypothesis 3: resilience had a significant negative effect on personal burnout (β = −0.49, *p* < 0.001) and a significant positive effect on work engagement (β = 0.16, *p* < 0.05).

With regard to Hypothesis 4, both interaction terms were found to be non-significant (Quantitative demands*Resilience: β = 0.07, SE = 0.05, *p* = 0.10; Emotional demands*Resilience: β = −0.07, SE = 0.05, *p* = 0.12). Resilience did not moderate the relationship between quantitative and emotional demands and personal burnout, so that Hypothesis 4 was rejected.

## 4. Discussion

To our knowledge, this study is the first to assess the job demands and resources of social workers serving refugees and homeless individuals by testing their predictive value for social workers’ burnout and work engagement in a structural equation model. We were able to gain important new insights into these topics based on reliable statistical methods.

### 4.1. Working Conditions of Social Workers in Refugee and Homeless Aid

In comparison with a norm sample of social and educational professions in Germany [42] as well as with the results of a recent interview study with social workers [8], respondents of the present study described similar patterns with regard to the measured variables. Substantial correlations between social workers’ job demands, resources, burnout, and work engagement were revealed, which are consistent with the propositions of the JD-R model [33,34]. Some results of the structural model provided support for the JD-R model as well, although not all of the proposed hypotheses were verified.

An important finding is that resilience as a personal resource had direct effects both on personal burnout and work engagement. The result is consistent with previous studies in which resilience showed negative effects on burnout and positive effects on work engagement [43,44,45]. In general, resilient people believe that they are able to solve problems on their own, which strengthens their perceived ability to act and contributes to increased motivation and engagement. Referring back to its definition, resilience enables flexible adaption to stressors and adverse situations [31,32]. Thus, possessing this personal resource may be particularly helpful in settings characterised by frequent changes and daily stressors, such as social work institutions [8]. However, no moderating effects of resilience could be found, meaning that, regardless of social workers’ levels of resilience, quantitative and emotional demands were associated with burnout. The findings thereby confirm the health impairment process suggested by the JD-R model [33,34]. Contrary to our hypotheses, the job demands did not affect work engagement. 

In accordance with a qualitative study showing that employees in social work reported high demands but simultaneously showed a strong motivation and commitment to their work [8], the present data confirmed meaning of work as an important job resource with a strong positive effect on work engagement. The finding is consistent with the preposition of the JD-R model, stating that job resources play an important role in fostering employees’ work engagement [33,34]. The phenomenon of simultaneously experiencing high demands and a strong sense of commitment could particularly apply to social workers and should be taken into consideration for the development of health promotion measures for this target group.

In contrast to previous results from varying occupations [21,46], social support did not show any direct effects on the studied outcomes in the structural model (although significant associations on a correlation level were found). In accordance with this, Kim and Stoner also found no evidence for a significant negative relationship of social support and burnout [47]. Several reasons for the non-significant effect of social support in the structural model could be discussed, which may partly relate to methodological issues. For example, the finding could be linked to the variable’s operationalization: While the other variables in the structural model refer to personal or inner evaluations of work and demands imposed on the individual, social support more strongly represents interactions with others. Thus, it could be assumed that social support rather has a moderating influence in buffering the impact of job demands on the workers’ health, which was also shown in previous research [46]. Moreover, the result might be linked to the difference in terms of how workers felt supported by their colleagues as opposed to their supervisors (the social support scale encompasses items for both groups). Respondents in our sample rated the support from their colleagues (*M* = 79.01) significantly higher than the support from their supervisors (*M* = 64.32). However, we did not assess the reasons why employees felt more or less supported by these entities. In future research, it would be interesting to examine how social support provided by workers at different hierarchical levels (supervisors and colleagues) impacts differently on health outcomes.

### 4.2. Strengths and Limitations

The strengths of our study are the systematic recruitment process, good rate of completed surveys from the participants, and use of advanced statistical modelling techniques. SEM combines the strengths of regression analysis, path analysis, and confirmatory factor analysis. Moreover, we used well-validated instruments that have previously shown strong validity and high internal consistency, as well as performed thorough reliability and validity checks prior to testing our models.

However, the cross-sectional study design does not allow to draw causal conclusions, and reverse causality in the relationships between the variables cannot be completely ruled out. Furthermore, data was assessed by self-report measures only, which may favour bias to the data. Another limitation could be that several institutions refused to participate in the study, while only few of them provided explanations for non-participation (e.g., not enough time due to high workloads). Since we were not able to assess potential differences between institutions who agreed and refused to participate, a potential non-response bias cannot be ruled out completely. Although a broad range of social workers (job location, type, and sponsor of institution) has been studied, representativeness across all social workers serving refugees and homeless individuals cannot be assumed.

### 4.3. Implications for Future Research and Practice

With respect to research implications, future studies should apply longitudinal research designs in order to examine short- and long-term dynamics between the variables studied and establish causality regarding their relationships. Furthermore, the influence of social support could be examined more closely and with regard to its moderating effects.

In terms of practical implications, our results indicate a need for the development of health promotion measures for social workers in homeless and refugee aid. It is recommended to implement both behavioural and structural workplace health promotion measures. Structural approaches should specifically target the reduction of employees’ job demands in order to diminish their potentially health-depleting effects. Since both emotional and quantitative demands had direct effects on burnout, which were not buffered by personal resource resilience, implies that employees’ job demands need to be targeted directly and reduced by appropriate measures (e.g., in terms of diminished workloads, changes in work tasks and hours, and additional staff). In addition, behavioural measures should be taken to foster employees’ resources. It seems especially valuable to strengthen social workers’ meaning of work and resilience, since both resources showed beneficial effects on employees’ work engagement and were negatively related to burnout. This could be done, for example, by offering tailored education, training, and counselling to the workers on different topics, such as resilience and self-care. Moreover, thematic workshops within or across teams could provide employees with a platform to reflect and become aware of their professional achievements, which could further strengthen their meaning of work. This could also help the workers to visualize the societal relevance of their activities and promote their job motivation and commitment.

## 5. Conclusions

The present study offers valuable insights into the working conditions and strain experienced by social workers in refugee and homeless aid. The results confirmed substantial associations between job demands, resources, burnout, and work engagement, and mainly supported the proposed mechanisms of the JD-R model. Although resilience did not moderate the link between employees’ job demands and personal burnout, its direct effects on burnout and work engagement are noteworthy. Overall, our study demonstrates a need for the development of health promotion measures in order to reduce social workers’ demands and foster their personal and job resources. For only healthy social workers are able to fulfil their crucial societal tasks.

## Figures and Tables

**Table 1:** Participant characteristics.

	*n*	% ^a^
Gender	240	
Female	165	68.8
Male	75	31.3
Age	243	
≤24 years	1	0.4
25–34 years	72	29.6
35–44 years	66	27.2
45–54 years	51	21.0
≥55 years	53	21.8
Work area	242	
Homeless aid	120	49.6
Refugee aid	94	38.8
Both areas	28	11.6
Professional qualification	246 *	
Social worker	153	62.2
Educator	6	2.4
Social care worker/remedial therapist	4	1.6
Humanities scholar	23	9.3
Law, economics and social sciences	45	18.3
Health-related apprenticeship	8	3.3
Business-related apprenticeship	4	1.6
Other	3	1.2
Work experience in social work	242	
0–3 years	65	26.9
4–10 years	79	32.6
11–20 years	37	15.3
>20 years	61	25.2
Type of institution	235	
Outpatient counselling centre	53	22.6
Day care centre, overnight accommodation	21	8.9
Initial registration centre	3	1.3
Shared accommodation, residential/transition home	97	41.3
Outpatient assisted living	48	20.4
Street social work, street magazine	3	1.3
Emergency shelter	3	1.3
Management, coordination, head office	7	3.0
Sponsor of institution		241
Independent sponsor (non-profit, charity, church)	181	75.1
Public sponsor	57	23.7
Commercial sponsor (profit-oriented)	3	1.2
Federal state		243
Berlin	93	38.3
Hamburg	90	37.0
Mecklenburg-Western Pomerania	24	9.9
Schleswig-Holstein	36	14.8

Note: ^a^ Percentages do not account for missing values. * Multiple choice answer.

**Table 2:** Characteristics of the variables.

Variables	Mean	SD	Range	Minimum	Maximum	α
**Quantitative demands**	53.96	17.38	0–100	0	100	0.79
**Emotional demands**	66.24	15.97	0–100	8.33	100	0.77
**Meaning of work**	81.40	17.42	0–100	16.67	100	0.85
**Social support**	72.76	20.51	0–100	12.50	100	0.81
**Work engagement**	68.74	11.17	0–100	0	100	0.84
**Personal burnout**	48.00	20.30	0–100	3.83	100	0.91
**Resilience**	61.32	18.97	13–91	32.00	90	0.88

Note: α = Cronbach’s Alpha.

**Table 3:** Pearson correlation coefficients for all variables.

Variables	Quantitative Demands	Emotional Demands	Meaning of Work	Social Support	Work Engagement	Personal Burnout	Resilience
**Quantitative demands**	−						
**Emotional demands**	0.37 ***	−					
**Meaning of work**	0.08	0.03	−				
**Social support**	−0.24 ***	−0.16 *	0.22 **	−			
**Work engagement**	−0.02	−0.15 *	0.68 ***	0.22 **	−		
**Personal burnout**	0.38 ***	0.44 ***	−0.24 ***	−0.22 ***	−0.41 ***	−	
**Resilience**	−0.05	−0.22 ***	0.34 ***	0.16 *	0.43 ***	−0.55 ***	−

Note: Pearson correlation coefficient: * *p* < 0.05; ** *p* < 0.01; *** *p* < 0.001.

**Table 4:** Reliability and validity analysis.

Variables	CR	AVE	√ AVE	Correlations
**Quantitative demands**	0.71	0.55	0.74	−0.24–0.45
**Emotional demands**	0.78	0.54	0.73	−0.25–0.50
**Meaning of work**	0.85	0.66	0.81	−0.26–0.79
**Social support**	0.79	0.52	0.72	−0.24–0.25
**Work engagement**	0.85	0.66	0.81	−0.15–0.79
**Personal burnout**	0.91	0.63	0.79	−0.63–0.50
**Resilience**	0.88	0.37	0.61	−0.63–0.47

Note: CR = composite reliability; AVE = average variance extracted, √ AVE = square root of the average variance extracted; Correlations = correlations between the latent variables.

**Table 5:** Standardized path coefficients.

	Personal Burnout				Work Engagement			
	β	(95% CI)	SE	*p*	Β	(95% CI)	SE	*p*
**Quantitative demands**	0.28	(0.14; 0.410	0.09	0.002	−0.04	(−0.18; 0.090	0.08	0.54
**Emotional demands**	0.26	(0.13; 0.40)	0.08	<0.001	−0.13	(−0.26; 0.02)	0.08	0.08
**Meaning of work**	−0.14	(−0.28; −0.01)	0.06	0.03	0.74	(0.59; 0.87)	0.12	<0.001
**Social support**	0.01	(−0.12; 0.14)	0.08	0.85	0.02	(−0.13; 0.15)	0.09	0.86
**Resilience**	−0.49	(−0.60; −0.35)	1.54	<0.001	0.16	(0.02; 0.29)	1.27	0.02
Quantitative demands * Resilience	0.07	(−0.01; 0.16)	0.05	0.10	−	−	−	−
Emotional demands * Resilience	−0.07	(−0.16; 0.02)	0.05	0.12	−	−	−	−

Note: β = standardized regression weight, 95% CI = 95% confidence interval (lower bound, upper bound), SE = standard error. *p* = *p*-values: * *p* < 0.05.
